# Molecular survey and phylogenetic analysis of *Bartonella* sp., *Coxiella* sp., and hemoplamas in pudu (*Pudu puda*) from Chile: first report of *Bartonella henselae* in a wild ungulate species

**DOI:** 10.3389/fvets.2023.1161093

**Published:** 2023-11-16

**Authors:** Ezequiel Hidalgo-Hermoso, Paulina Sepúlveda-García, Javier Cabello, Sebastian Celis, Carola Valencia, Carolina Ortiz, Ignacio Kemec, Dario Moreira-Arce, Miguel Orsola, Nivia Canales, Antonio Garnham, Frank Vera, Ananda Muller

**Affiliations:** ^1^Fundacion Buin Zoo, Buin, Chile; ^2^Instituto de Medicina Preventiva Veterinaria, Facultad de Ciencias Veterinarias, Universidad Austral de Chile, Valdivia, Chile; ^3^Escuela de Graduados, Facultad de Ciencias Veterinarias, Universidad Austral de Chile, Valdivia, Chile; ^4^Centro de Conservación de la Biodiversidad, Chiloé-Silvestre, Ancud, Chiloé, Chile; ^5^Departamento de Veterinaria, Parque Zoológico Buin Zoo, Buin, Chile; ^6^Facultad de Ciencias de la Naturaleza, Sede De La Patagonia, Universidad San Sebastián, Puerto Montt, Chile; ^7^Departamento de Gestión Agraria, Universidad de Santiago de Chile (USACH), Santiago, Chile; ^8^Institute of Ecology and Biodiversity (IEB), Santiago, Chile; ^9^Centro Integrativo de Biología y Química Aplicada (CIBQA), Universidad Bernardo O'Higgins, Santiago, Chile; ^10^Instituto de Bioquímica y Microbiología, Facultad de Ciencias, Universidad Austral de Chile, Valdivia, Chile; ^11^Escuela de Medicina Veterinaria, Universidad Mayor, Santiago, Chile; ^12^Biomedical Sciences Department, Ross University School of Veterinary Medicine, Basseterre, Saint Kitts and Nevis; ^13^Instituto de Ciencias Clínicas Veterinarias, Facultad de Ciencias Veterinarias, Universidad Austral de Chile, Valdivia, Chile

**Keywords:** zoonotic diseases, wildlife host, endangered species, *Coxiella burnetii*, hemoplasmas

## Abstract

**Introduction:**

Recent evidence shows a high diversity of infectious agents in wildlife that represent a threat to human, domestic, and wild animal health. In Chile, wild populations of the most common cervid species, pudu (*Pudu puda*), have been reported as hosts for novel pathogens such as *Mycoplasma ovis*-like and a novel ecotype of *Anaplasma phagocytophilum*. A better understanding of the epidemiology of this group and other intracellular bacteria that might have cervids as hosts would enlighten their population relevance. This study aimed to determine the occurrence and genetic diversity of *Bartonella* spp., hemotropic mycoplasmas, and *Coxiella burnetii* in pudus from Chile.

**Methods:**

The DNA was extracted from the blood samples of 69 wild free-ranging and 30 captive pudus from Chile. A combination of real-time (nouG gene for *Bartonella* and IS1111 element for *C. burnetii*) and conventional PCR (16S rRNA for hemotropic *Mycoplasma* spp. and rpoB, gltA, and ITS for *Bartonella* spp.) was used for pathogen screening and molecular characterization.

**Results:**

DNA of *Bartonella* spp. was detected in 10.1% [95% CI (5.2–18.2%)] samples, hemotropic *Mycoplasma* spp. in 1.7% [95% CI (0.08–10.1%)], and *C. burnetii* in 1.0% [95% CI (0.05–6.3%)] samples. Two sequenced samples were identified as *Mycoplasma ovis*-like, and one free-ranging pudu was positive for *C. burnetii*. While one captive and two free-ranging pudus were positive for *Bartonella henselae*, one wild pudu was co-positive for *B. henselae* and *Bartonella* sp., similar to *Bartonellae* identified in ruminants.

**Discussion:**

To the best of our knowledge, this is the first report of *B. henselae* in wild ungulate species, and *C. burnetii* and *Bartonella* spp. in wild ungulate species in South America. Further research will be necessary to evaluate the potential role of pudu as reservoirs of infection and identify the sources for disease transmission among humans and wild and domestic animals.

## 1 Introduction

The recognition of the role of wildlife as reservoirs of pathogens that threaten the health of humans and/or livestock species has increased in the past several decades; accordingly, the relevance of infectious agents in the wildlife conservation field has also amplified (1, 2). As expected, there are differences between regions of the world. In South America, for example, there is a lack of scientific publications on infectious diseases in wildlife when compared with more developed countries (3–6).

*Bartonella* spp., *Coxiella burnetii*, and hemotropic *Mycoplasma* spp. are intracellular bacteria that infect a wide range of animals (7–9) and humans. Hemotropic mycoplasmas (hemoplasmas) are obligate epi-erythrocytic, cell wall-deficient bacteria that usually generate hemolytic anemia in numerous animal species. Routes of transmission are not fully elucidated, but aggressive interactions and possibly fleas and ticks might be involved. The pathogenic potential of hemotropic mycoplasmas, as a cause of human disease, has not been clearly defined; the public health implications derived from these emerging zoonotic pathogens are underestimated (10). *Bartonella* is composed of gram-negative fastidious, facultative intracellular microorganisms transmitted by fleas and other vectors that provoke a long-lasting bacteremia in the mammal host. The zoonotic potential of these bacteria is well described, and the term bartonellosis has been implemented to refer to human diseases (11). *Coxiella burnetii* is a zoonotic, strictly intracellular gram-negative bacterium that infects a wide range of animals. In its sylvatic cycle, it can be transmitted by ticks. In humans, it is considered the causal agent of query fever (Q-fever), and the Centers for Disease Control and Prevention (CDC) has classified this microorganism as a potential bioterrorism agent (12).

There is increased evidence that wildlife species are also susceptible (13–15) and have the potential to be zoonotic (7, 16, 17). In Chile, several domestic and wildlife species have been identified as potential hosts for several hemoplasmas (18–22) and *Bartonella* spp. (23–28). Information on *Coxiella burnetii* is much more limited, with only one report with molecular evidence in bats from Chile (26). Despite being commonly reported in domestic and wild ruminants in Europe and North America (13, 29, 30), there are no studies for the detection of *Bartonella* spp. and *C. burnetii* in these taxa in Chile, and only until recently has it been possible to identify hemoplasmas in domestic camelids, llamas (*Lama glama*), and alpacas (*Vicugna pacos*) (31). Finally, the native pudu (*Pudu puda*) has been identified as the potential host species of several hemoplasmas (10), including *Mycoplasma ovis*-like, in the Chilean template forest.

Pudu is the most common cervid in Argentina and Chile and is considered threatened in both countries (32, 33), as shown in CITES Appendix I. In Chile, pudus inhabit temperate forests heavily affected by anthropic factors such as deforestation, housing construction, free-ranging dogs, and livestock (34). Additionally, a high diversity of infectious agents that could be a threat to their health status has recently been identified (10, 35–38). Recently, pudus were identified as potential reservoir hosts for the bovine viral diarrhea virus, which is a cause of major disease in cattle (39). This study aimed to determine the occurrence and genetic diversity of *Bartonella* spp., hemotropic mycoplasmas, and *C. burnetii* in free-ranging and captive pudus from Chile.

## 2 Materials and methods

### 2.1 Animal sampling

Blood samples from frozen banks in rescue centers and zoos/breeding centers were used. The frozen bank samples were opportunistically collected from 69 free-ranging pudus between 2016 and 2022 on admission day from two wildlife rehabilitation centers in the template forest ecosystem of southern Chile in Los Lagos District, one (USS: Cerefas, Universidad San Sebastian) located in the continental area and the other (Ch. S: Chiloe Silvestre NGO) in Chiloe island. Additionally, blood samples from 30 captive pudus were collected between 2017 and 2021 during preventive medicine procedures in two facilities, one located in the Mediterranean ecosystem of Central Chile in the Metropolitan District and the other in Los Lagos District, and do not have contact between centers. Blood samples were obtained by venipuncture of the jugular vein using an evacuated tube system (Vacutainer, Beckon, Dickson and Company, Franklin Lakes, New Jersey, USA) and stored at −20°C within 6 h of collection. For extensive sampling details, refer to the study mentioned in the reference (10).

### 2.2 Molecular detection and phylogenetic analysis

#### 2.2.1 DNA extraction/purification

The 99 frozen EDTA-blood samples were thawed at room temperature and vortexed at the UACh Veterinary Clinical Pathology Laboratory, Valdivia, Chile. DNA extraction from 200 μl of blood was performed using an E.Z.N.Z. Tissue DNA Kit (E.Z.N.A. Omega BioTek^®^, Norcross, GA, U.S.A.), according to the manufacturer's instructions, to obtain a concentration between 20 and 50 ng/μl of purified DNA. Concentration and purity of DNA were measured (NanoDrop ND-1000, Thermo Scientific, Waltham, MA, U.S.A.). The 260/280 nm absorbance ratio (OD_260_/OD_280_) provided an estimate of sample purity, accepting a ratio of 1.8 ± 0.2 as pure. DNA was stored at −20°C before performing PCR assays.

#### 2.2.2 Endogenous control conventional (c) PCR

DNA samples were subjected to qPCR targeting the irbp gene (interphotoreceptor retinoid-binding protein) using the primers IRBP-CF_FWD (5′-TCCAACACCACCACTGAGATCTGGAC-3′) and IRBP-CF-REV (5′-GTGAGGAAGAAATCGGACTGGCC-3′), with the aim to check DNA template integrity and discard the presence of PCR inhibitors, as previously described (40). All cPCRs were performed with nuclease-free water as a negative control in a T100TM Thermal Cycler (Bio-Rad).

#### 2.2.3 Quantitative real-time PCR for *Bartonella* spp. screening

To detect and quantify *Bartonella* spp., the DNA of all irbp cPCR-positive samples were subsequently subjected to an initial screening by quantitative real-time PCR (qPCR) targeting the *nuoG* gene of *Bartonella* spp. (83 bp), using primers (F-Bart [5′-CAATCTTCTTTTGCTTCACC-3′] and R-Bart [5′- TCAGGGCTTTATGTGAATAC-3′], hydrolysis probe (TexasRed-5′- TTYGTCATTTGAACACG-3′[BHQ2a-Q]3′) as previously described (41). qPCR amplifications were conducted in Hard-Shell PCR plates (Bio-Rad©, CA, USA) using Thermal Cycler CFX96 Touch Real Time (Bio-Rad, CA, USA). Amplification efficiency (E) was calculated from the standard curve slope in each run using the following formula: (E = 10–1/slope). Copy numbers were estimated using 10-fold serial dilutions of gBlock^®^ (Integrated DNA Technologies, Coralville, IA, U.S.A.), encoding the *nuoG B. henselae* sequence (insert containing 83 bp). *Bartonella henselae* genomic DNA from a cat tested in a previous study was used as a positive control (42). All PCR runs were performed with nuclease-free water (Promega^®^, Madison, WI, USA) as a negative control. Replicates showing a Cq difference higher than 0.5 were retested.

#### 2.2.4 Conventional (c) PCR for *Bartonella* spp. characterization

All positive *Bartonella* spp. *nuoG*-qPCR positive samples were subjected to cPCR amplification of a fragment of three loci [*gltA* (43), *rpoB* (44), and ITS (45)] by cPCR with the aim to molecularly characterize *Bartonella* spp. cPCR amplification reactions were performed in a T100 Bio-Rad thermocycler (Bio-Rad©, Hercules, CA, U.S.A.), and the details of the amplification conditions are presented in Table 1. *Bartonella henselae* genomic DNA from a cat tested in a previous study was used as a positive control (42).

**Table 1:** Summary information of the conventional and Real time PCR primer sets, amplification conditions and their amplicon sizes used in the present study.

**Target**	**Primers**	**Amplification cycles**	**Amplicon size (pb)**	**Reference**
**Endogenous control**				
**Interphotoreceptor Retinol-Binding Protein (IRBP)**	IRBP-CF_FWD (5′-TCCAACACCACCACTGAGATCTGGAC-3′) IRBP-CF-REV (5′-GTGAGGAAGAAATCGGACTGGCC-3′)	95°C × 4min 94°C × 30s 52°C × 30s 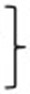 35cycles 72°C × 1 min 72°C × 5 min	227	(40)
**Screening real time PCR**				
**Nicotinamide adenine dinucleotide dehydrogenase gamma subunit (NUOG) gene of** ***Bartonella*** **spp**.	F-Bart (5′-CAATCTTCT TTTGCTTCACC-3′) R-Bart (5′-TCAGGGCTTTAT GTGAATAC-3′) Hydrolysis probe: TexasRed-5′-TTYGTCATTTGAACA CG-3′[BHQ2a-Q]3′	95°C × 3 min 95°C × 10 min 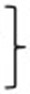 40 cycles 52.8°C × 30s	83	(41)
**Multicopy insertion sequence (Is111) of** ***Coxiella Burnetii***	Cox-F: (5′-GTCTTAAGGTGGGCTGCGTG-3′) Cox-R: (5′-CCCCGAATCTCATTGATCAGC3′) Hydrolysis probe: FAM-5′-AGCGAACCATTGGTATCGGACGTT- 3′TAMRA-TAT GG	50°C × 2 min 95°C × 10 min 95°C × 15s 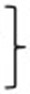 45 cycles 60°C × 30s	295	(46)
**Conventional PCR molecular characterization**				
**Citrate synthase (*****GLTA*****) gene of** ***Bartonella*** **Spp**.	CS443f (5′-GCTATGTCTGCATTCTATCA -3′) CS1210r (5′- GATCYTCAATCATTTCTTTCCA -3′)	94°C × 2 min 94°C × 30s 48°C × 1 min 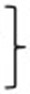 45 cycles 72°C × 1 min 72°C × 5 min	767	(43)
**Intergenic tegion 16s-23s rRNA (ITS) of** ***Bartonella*** **Spp**.	325s (5′-CTTCAGATGATGATCCCAAGCCTTYTG GCG -3′) 1100as (5′- GAACCGACGACCCCCTGCTTGCAAAGC A-3′)	95°C × 5 min 94°C × 15s 66°C × 15s 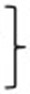 55 cycles 72°C × 15s 72°C × 1 min	453- 717	(45)
**β** **subunit of rna polymerase (*****RPOB*****) of** ***Bartonella*** **spp**.	rpoBF (5′-GCACGATTYGCATCATCATTTTCC-3′) rpoBR (5′-CGCATTATGGTCGTATTTGTCC-3′)	95°C × 5 min 94°C × 45s 52°C × 45s 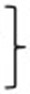 40 cycles 72°C × 45s 72°c × 7 min	333	(44)
**16s** ***rRNA*** **gene of Haemotropic** ***Mycoplasma*** **spp**.	HemMyco16S-322s: GCCCATATTCCTACGGGAAGCAGCAGT HemMyco16S-938as: CTCCACCACTTGTTCAGGTCCCCGTC	95°C × 5 min 94°C × 15s 68°C × 15s 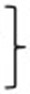 55 cycles 72°C × 18s 72°C × 30s	620	(45)

#### 2.2.5 Quantitative real-time PCR for Coxiella burnetii screening

The screening real-time qPCR targeted a 295-bp fragment of the multicopy insertion element IS1111 and is used for sensitive detection of *C. burnetii* in biological samples (46) (Table 1). Primers Cox-F (GTC TTA AGG TGG GCT GCG TG) and Cox-R (CCC CGA ATC TCA TTG ATC AGC) and hydrolysis probe Cox-TM (FAM-AGC GAA CCA TTG GTA TCG GAC GTT–TAMRA–TAT GG) were used. Standard curves were constructed using 10-fold serial dilutions (2.0 × 10^7^ to 2.0 × 10^0^) of a gBlock^®^ (Integrated DNA Technologies, Coralville, IA, USA), encoding a 295-bp fragment of the IS1111 element of *C. burnetii* (Integrated DNA Technologies, Coralville, IA, USA). Amplification efficiency (E) was calculated from the standard curve slope in each run using the following formula (E = 10–1/slope). *Coxiella burnetii* genomic DNA from a cow was used as a positive control. All PCR runs were performed with nuclease-free water (Thermo Scientific©, Waltham, MA, USA) as a negative control. Replicates showing a Cq difference higher than 0.5 were retested.

#### 2.2.6 Conventional (c) PCR for hemotropic *Mycoplasma* spp.

All positive samples in the irbp cPCR were subjected to a cPCR protocol targeting the 16S rRNA hemotropic *Mycoplasma* spp. gene (620 bp), using HemMycop16S-322s and HemMycop16S-938as primers (Table 1), according to a previously described protocol (7). All cPCR runs were performed with nuclease-free water (Thermo Scientific©) as a negative control, and a cat sample known to be infected by *M. haemofelis* was used as a positive control. This protocol was used for screening and later sequencing for molecular characterization of detected hemoplasmas.

#### 2.2.7 Electrophoresis

Conventional PCR products were separated by 1.5% agarose gel electrophoresis (LE Agarose Seakem^®^, Lonza) and stained with SYBR© safe DNA gel stain (Thermo Scientific©). The DNA products with the expected size were purified and sequenced.

#### 2.2.8 Purification and sequencing

Only positive samples presenting strong band intensity (*Bartonella* spp. and hemotropic *Mycoplasma* spp.) were purified by enzymatic reaction using ExoSAP- IT^TM^ PCR Product Cleanup Reagent (Thermo Scientific©, Carlsbad, CA, U.S.A.), following the manufacturer's instructions. Purified DNA was sent to MACROGEN (Seoul, Korea) for sequencing by the Sanger method in an automatic sequencer (A.B.I Prism 310 genetic analyzer; Applied Biosystem ©/PerkinElmer) for species identification. Forward and reverse sequences were analyzed in Geneious 7.1 (https://www.geneious.com), to obtain consensus sequences. Identity percentages were obtained using BLASTn (47).

### 2.3 Phylogenetic analysis

Before constructing the phylogenetic inference, sequences belonging to different samples, but representing the same bacterial species, were aligned with Geneious 7.1 (https://www.geneious.com) using the MAFFT alignment method (48) and subsequently analyzed for detection of polymorphism and haplotype identification using DnaSP v5 software (49).

The sequences of the present study were aligned with other sequences from the database (GenBank) through the MAFFT program (Multiple Alignment by Fast Fourier Transform) (48) incorporated in Geneious 7.1 software (https://www.geneious.com). Then, multiple alignments were analyzed using BMGE (Block Mapping and Gathering with Entropy) software to remove ambiguously aligned regions (50).

For the phylogenetic analysis, the best evolutionary model was selected according to the Bayesian Information Criterion (BIC) for each one of the codon positions (partition) for the encoded genes (*gltA* and *rpoB*) (51). Thus, the best evolutionary models for *Bartonella* spp. *gltA* were K3P+G4 (partition 1), TIM3+F+G4 (partition 2), and TNe+G4 (partition 3). For *Bartonella* spp. *rpoB*, the best models were TPM3u+F+G4 (partition1), TNe+G4 (partition 2), and TIM3e+G4 (partition 3). For the non-coding genes (ITS), the best evolutionary model was selected according to the Akaike information criterion (AIC) (52). The best model for *Bartonella* spp. ITS was TPM2u+F+G4. For ITS, the best evolutionary model selection was assessed using Model Finder (53). Finally, all trees were inferred with a bootstrapping of 1,000 by the maximum likelihood (ML) method with IQ-TREE (54). To enroot the trees, the outgroups were the following for the *Bartonella* spp. trees: *Ochrobactrum anthropii (gltA*, ITS, *rpoB*), *Brucella abortus* (*gltA, rpoB*), and *Brucella melitensis* (ITS). *Mycoplasma pneumoniae* was used as an outgroup for the construction of the 16S rRNA hemoplasma tree.

## 3 Results

### 3.1 *Bartonella* spp. qPCR results

All DNA samples (median and standard deviation (SD) of DNA concentration = 31.5 ± 56.2 ng/uL; mean and SD 260/280 ratio = 1.3 ± 0.35) were positive for the *irbp* gene.

Molecular occurrence of *Bartonella* spp. DNA in pudu detected by qPCR (mean and SD of reactions' efficiency = 100 ± 5.04%; r2 = 0.99 ± 0.005; slope = −3.32 ± 0.11; Y-intercept = 39.26 ± 1.09) was 10.1% (10/99) [95% CI (5.2–18.2%)]. Only three samples had consistent Cq, and the quantification of *Bartonella* spp. was 18.5 ± 14.02 *nuoG*-copies/μl (mean ± standard deviation, SD).

Representative sequences of *Bartonella* spp. *gltA*, ITS, and *rpoB* genes were deposited in GenBank (55) under the accession numbers OQ162290, OQ137267, and OQ162291. Within sequences that represented the same haplotype, only one representative sequence (with a higher size) was deposited in GenBank and used for phylogenetic analysis.

#### 3.1.1 *Bartonella* spp. cPCR results

*Bartonella* spp. DNA was successfully amplified by cPCR in 60% (6/10) of qPCR-positive samples, and six sequences were obtained [4 *rpoB* (samples: #6235, #5144, #8184, and 902020), 1 *gltA* (sample #6235), 1 ITS (sample #6235)] from four pudus [one captive (902,020) and three free-ranging (6,235, 5,144, and 8,184)]. The *rpoB* sequences were 100% similar to each other and showed 98.2% similarity with *B. henselae* from cats in Brazil (MN107418), 99.7% identity with *B. henselae* from a cat from Paraguay (MW514669), and 100% identity with *B. henselae* from *Urva auropunctata* from St. Kitts (MW728257). The *gltA* sequence showed 95.05% identity with uncultured *Bartonella* sp. from a cattle tail louse from Israel (KJ522487), and the ITS sequence showed 93.1% identity with *Bartonella* sp. from deer ked (DQ485307). As such, wild pudu #6235 was co-positive to *B. henselae* and *Bartonella* sp., similar to Bartonellae identified in ruminants.

#### 3.1.2 *Bartonella* spp. phylogenetic analysis

**Figure 1:**
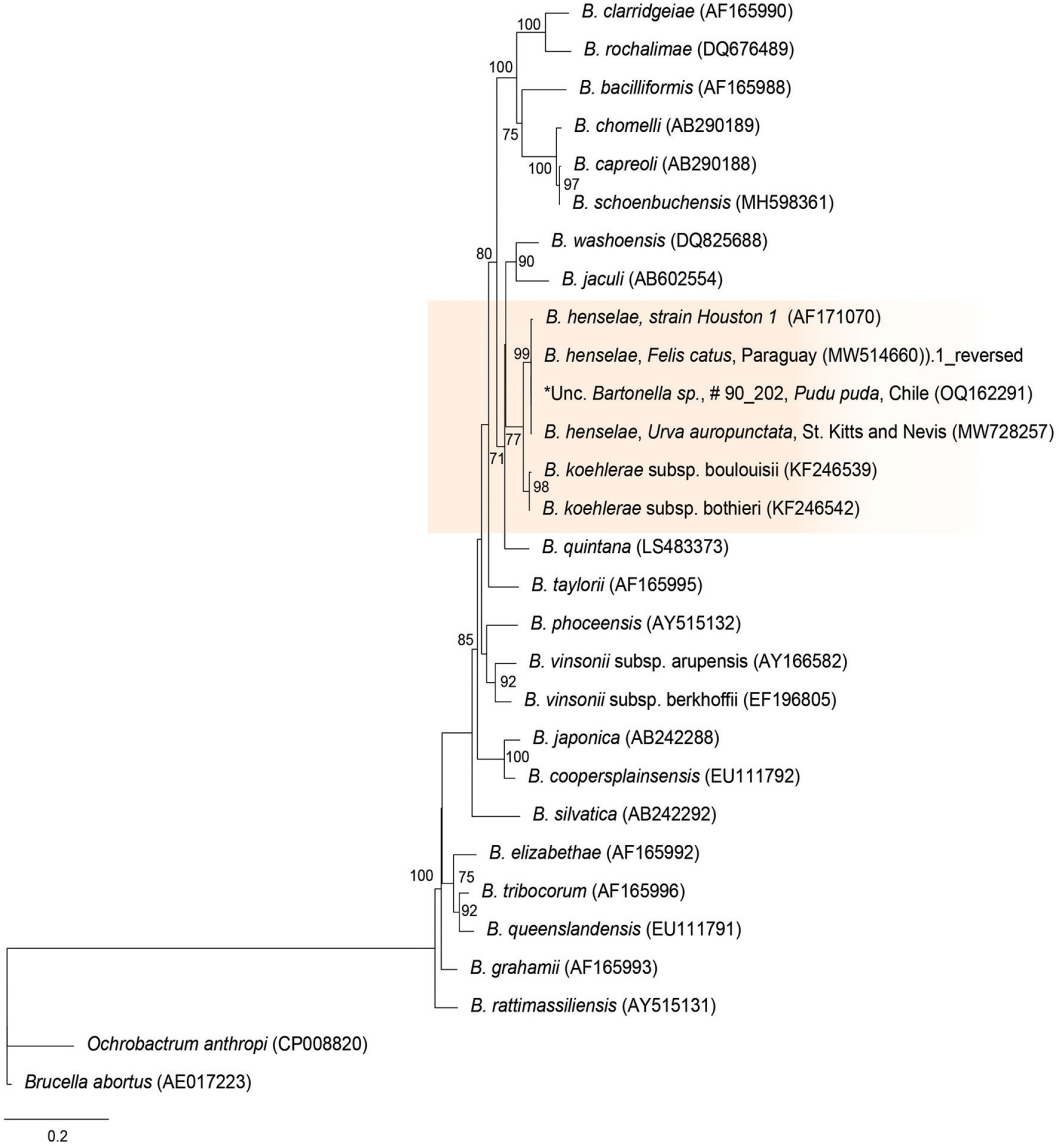
Maximum likelihood phylogenies for a subset of *Bartonella* spp. inferred using an alignment (1,152 bp) of the gene encoding the β subunit of RNA polymerase (*rpoB*). Calculated substitution model was TPM3u+F+G4 (partition 1), TNe+G4 (partition 2), and TIM3e+G4 (partition 3). Best models were chosen using the Bayesian information criterion (BIC).

The *rpoB* sequences of the present study were allocated in the same taxa, sharing a clade with *B. henselae* Houston 1 (AF171070), *B. henselae* from a cat from Paraguay (MW514660), and *B. henselae* from *Urva auropunctata* from St. Kitts and Nevis (MW728257) (Figure 1). The rpoB diversity analyses are represented on Table 2.

**Table 2:** Polymorphism and genetic diversity of *rpoB Bartonella* species sequences identified in pudu from Chile.

**Gene**	**bp**	**N**	**VS**	**GC%**	**H**	**Hd (mean ±SD)**	**Π (mean ±SD)**	**K**
*rpoB*	4	219	0	0.42	1	0	0	0

N, number of sequences analyzed; VS, number of variable sites; GC%, C + G content; h. number of haplotypes; hd, diversity of haplotypes; S.D., standard deviation; π, nucleotide diversity (per site); K, nucleotide difference number.

**Figure 2:**
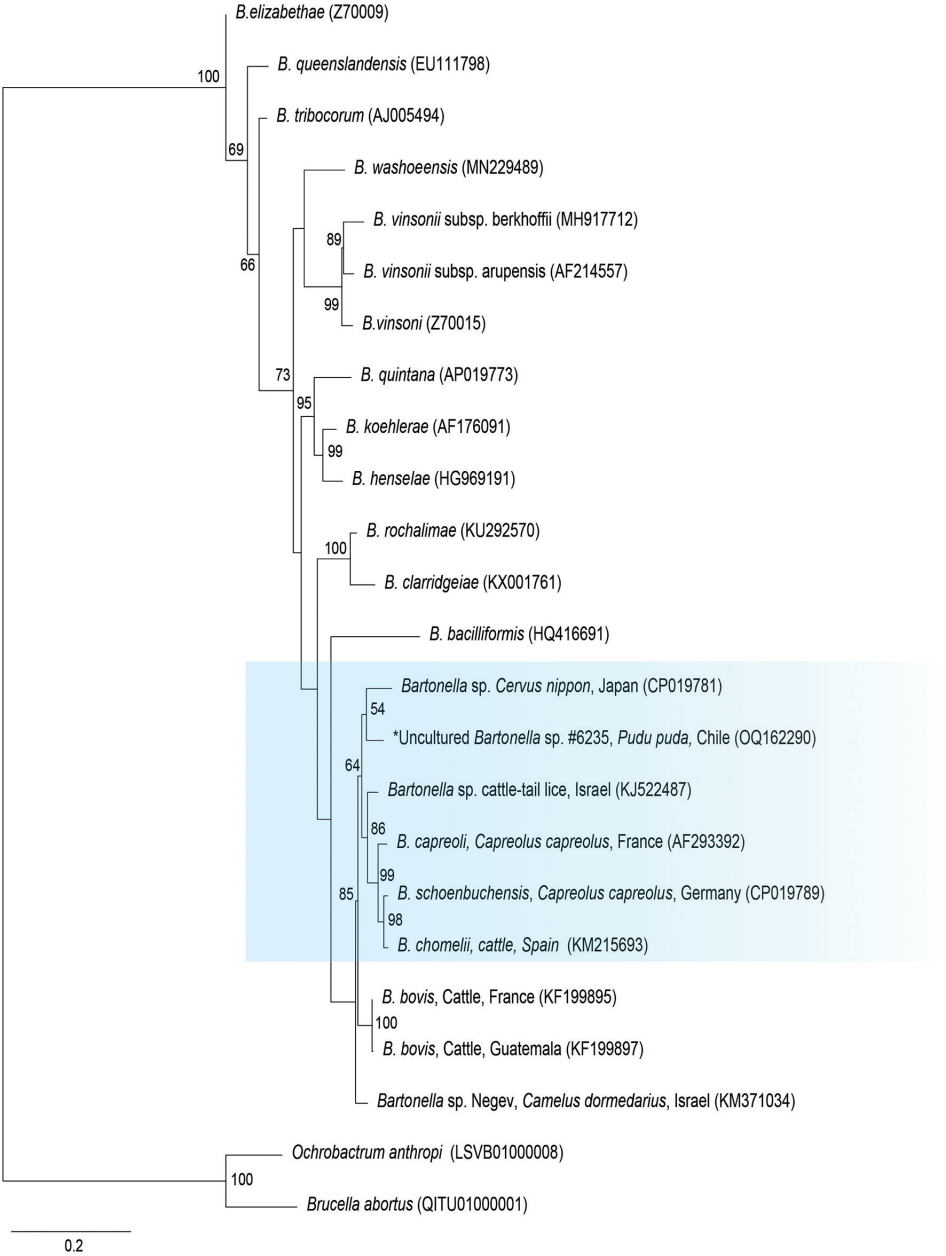
Maximum likelihood phylogenies for a subset of *Bartonella* spp. inferred using an alignment (1,290 bp) of the gene encoding citrate synthase (*gltA*). Calculated substitution model was K3P+G4 (partition 1), TIM3+F+G4 (partition 2), and TNe+G4 (partition 3). Best models were chosen using the Bayesian information criterion (BIC).

The *gltA* phylogenetic reconstruction evidenced that the sequence of the present study was allocated to the same clade with *Bartonella* sp. from a Cervus from Japan (CP019781), *Bartonella* sp. from a cattle tail louse from Israel (KJ522487), and *B. capreoli, B. schoenbuchensis*, and *B. chomeli* (Figure 2).

**Figure 3:**
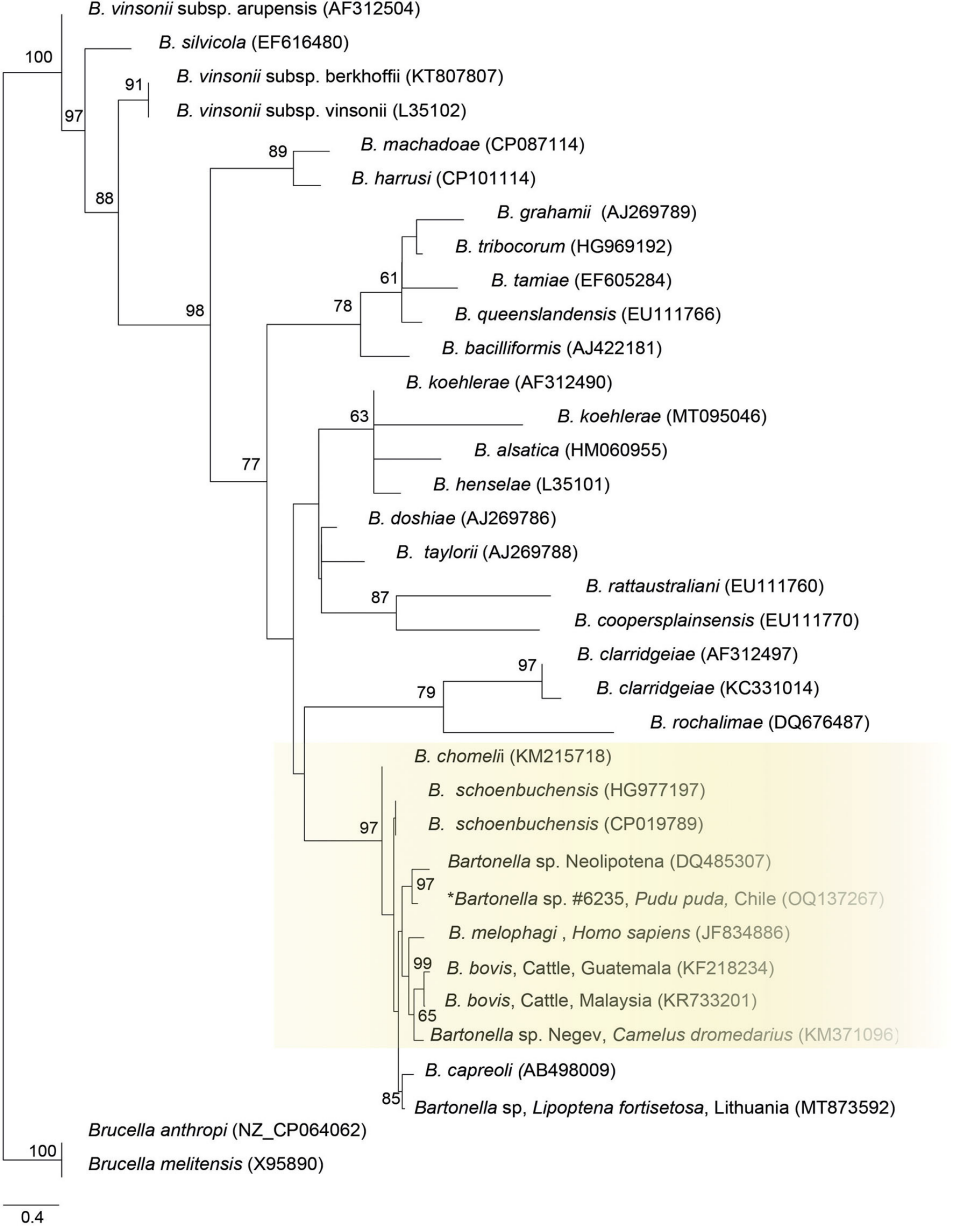
Maximum likelihood phylogenies for a subset of *Bartonella* spp. inferred using an alignment (522 bp) of the internal transcribed spacer (ITS). Calculated substitution model was TPM2u+F+G4. Best models were chosen using the Akaike information criterion (AIC).

Finally, the ITS sequence was closely positioned to *Bartonella* sp. sequence from a deer-ked (DQ485307), *B. schoenbuchensis* (CP019789, HG77197), *B. chomeli* (KM215718), *B. melophagi* (JF834886), and *B. bovis* (KF218234, KR733201) (Figure 3).

### 3.2 *Coxiella burnetii* qPCR results

Molecular occurrence of *C. burnetii* DNA in pudu detected by qPCR (mean and SD of reactions' efficiency = 100.6 ± 5.08%; r2 = 1.0 ± 0.005; slope = −3.31 ± 0.12; Y-intercept = 37.38 ± 0.88) was 1.0% (1/99) [95% CI (0.05–6.3%)].

#### 3.2.1 Hemotropic *Mycoplasma* cPCR results

**Figure 4:**
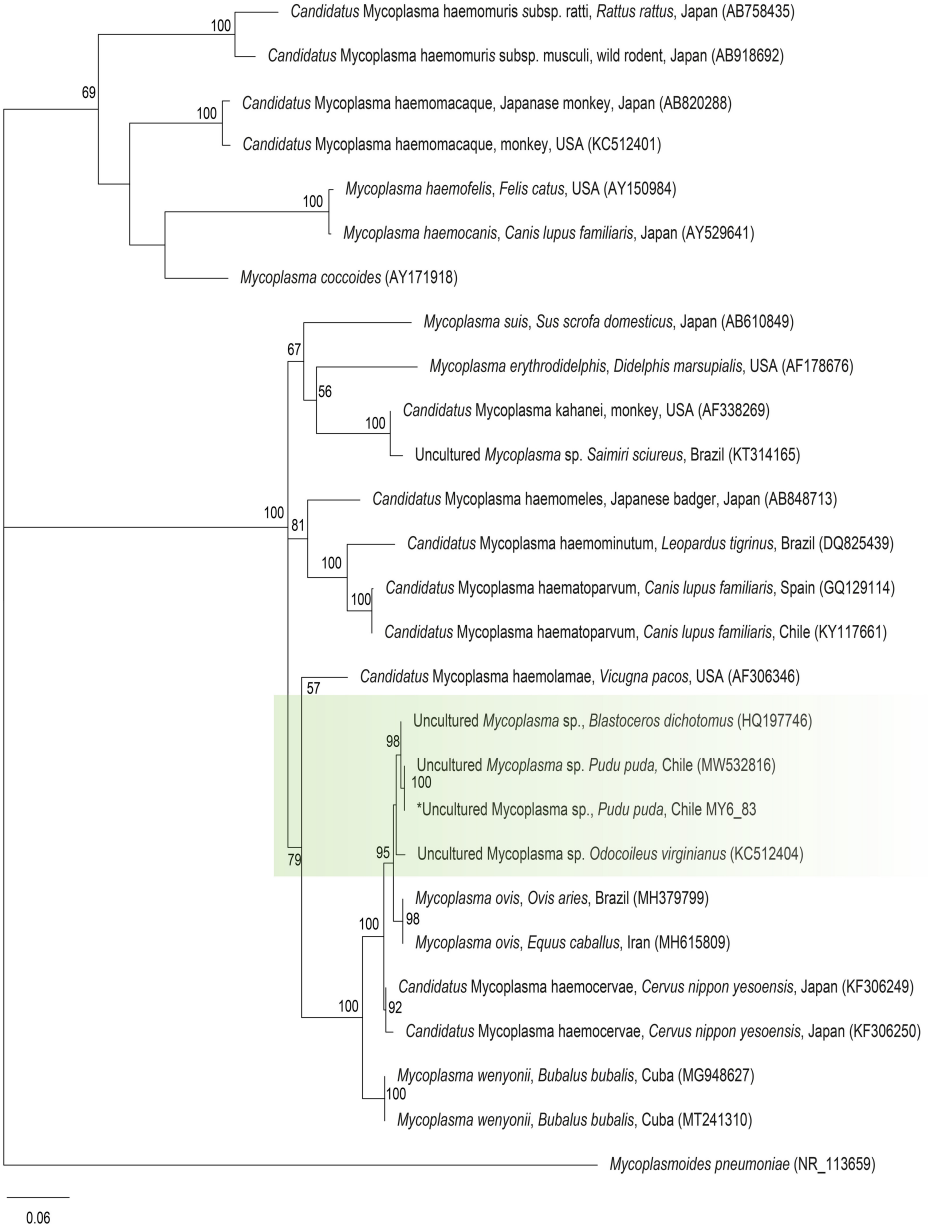
Maximum likelihood phylogenies for a subset of *Mycoplasma* spp. inferred using an alignment (620 bp) of the 16S rRNA gene. Calculated substitution model was GTR+F+G4. Best models were chosen using the Akaike information criterion (AIC).

Molecular occurrence of hemotropic *Mycoplasma* spp. in pudu by cPCR was 1.7% (1/60) [95% CI (0.08–10.1%)]. The sequence of the 16S rRNA fragment showed 100% BLASTn identity with *Mycoplasma ovis-*like amplified previously from Chilean pudu (MW532816) (Figure 4).

## 4 Discussion

This is the first study to document the presence of DNA of *B. henselae* in a wild ungulate species and *Bartonella* spp. and *C. burnetii* in wild ungulate species in South America. The circulation of *Mycoplasma ovis*-like in free-ranging pudu in Chile is also confirmed (10). The presence of these intracellular bacteria in free-living pudu could suggest an increase in the interaction between domestic species and their ectoparasites and these native species in their natural habitats. Unlike studies in wildlife in other regions (56–58), no co-infection with the three evaluated pathogens was found in pudus. However, one pudu was possibly co-infected with more than one *Bartonella* species, since *B. henselae* and *Bartonella* sp., similar to Bartonellae identified in ruminants, were detected. The co-occurrence of different *Bartonella* species in the bloodstream of reservoir animals such as pudus was earlier described in cats and rodents (59–61), and it illustrates the outstanding tolerance of these hosts to harbor mixed *Bartonella* infections. This could be mediated by an arthropod vector via multiplication and interaction of different *Bartonella* genetic variants in their digestive tract, with subsequent simultaneous transmission to the mammal host (62, 63). Culture and further molecular characterization of the isolates (64) should be attempted with these samples in future to confirm the co-positivity with multiple species of *Bartonella*.

*Coxiella burnetii* is an important bacterial zoonotic pathogen that can cause Q fever in humans. The bacterium has the potential to cause large-scale outbreaks due to its low infectious dose, environmental resistance, and ability to spread airborne through aerosolization of the pathogen, and is a potential biological threat classified as a “Select Agent” in the USA. *Coxiella burnetii* has a worldwide geographical distribution, apart from Antarctica and New Zealand, and has a wide and diverse host range. The pathogen primarily affects sheep, goats, and cattle, which are considered their primary reservoirs and sources for human outbreaks (65). The livestock species can be infected with *C. burnetii* and appear healthy, and people often become exposed by breathing in dust contaminated with animal feces, urine, and birth products. Wild ungulate species have been reported commonly exposed to *C. burnetii* infection in Europe and North America (66), including eight cervid species, but this report in pudu represents the first in deer from the Southern Hemisphere (65). In the Basque region in Spain, the prevalence has been categorized as stable throughout time. Therefore, the roe deer (*Capreolus capreolus*) plays a role in the sylvatic cycle of Q fever (67). In South America, there is no evidence of *C. burnetii* DNA in blood samples of wild boar (*Sus scrofa*), marsh deer (*Blastocerus dichotomus*), brown brocket deer (*Mazama gouazoubira*), small red brocket deer (*Mazama bororo*), red brocket deer (*Mazama americana*), and pampas deer (*Ozotocerus bezoarticus*) (12, 68). A recent study (12) found that 5.32% of the sampled deer was seropositive for *C. burnetii* by an indirect immunofluorescence assay (IFA) for IgG antibodies (anti-phase I); to date, it is the only evidence of exposure to this pathogen in deer in the region.

In Chile, DNA findings of *C. burnetii* have been reported in samples of animal origin only in bats and bulk tank milk from cows (26, 69). The last human Q fever outbreak in Chile was declared in 2017 in the Los Lagos District, the same region where molecular evidence was found in pudu in our study (70). This district is a part of the southern macrozone where seropositivity for humans (6%) was significantly higher than in other regions of the country (70). It is likely that the source of infection for pudu is of anthropogenic origin (livestock), or from exotic deer species, red deer, and/or fallow deer, which have been reported in the area (71) and have been commonly reported infected by *C. burnetii* in Europe (60, 65), or from rodent species previously found to be a source of livestock coxiellosis (72). Other serological or molecular studies in dogs and Darwin fox (*Lycalopex fulvipes*) in the southern macrozone found no evidence of *C. burnetii* infection (18, 73, 74). The finding of only one pudu being positive for the bacterium and the low prevalence of *C. burnetii* in Chile make serological and molecular screening necessary for a much larger number of pudu samples from the Los Lagos region, to evaluate their potential role as a host of infection for transmission to animals and humans. Additionally, the reports of infectious abortions in captive pudus in Chile (39) added to the evidence that *C. burnetii* has been involved in reproductive loss in captive exotic ungulates, mainly in bovid species (65), making it necessary to include in the differential diagnosis of possible causes of abortion in pudu. Moreover, future studies should attempt to molecularly characterize *via* sequencing the *C. bunetii* found in pudus from Chile.

In this study, *B. henselae*, an emerging zoonotic pathogen that causes scratch disease in humans and whose transmission mainly involves domestic cats as the main reservoir and cat fleas (*Ctenocephalides felis*) as the main vector (75), is described for the first time in wild ungulate species. Otherwise, to a lesser extent, it is reported in other mammals, bovines (76, 77) and rodents among them (78–81), suggesting that they have a permissive cycle in nature, being detected in several ecological niches (hosts and vectors) (77). Thus, this finding could indicate the circulation of *B. henselae* in an infected vector, favoring *B. henselae* transmission among domestic and wild mammals. Nonetheless, further epidemiological and genotyping studies are necessary to confirm this hypothesis. The DNA of *Bartonella* bacteria has been widely described in cervid species from Europe (30, 58, 82–84), North America (29, 85–87), and Asia (88, 89), usually with a higher prevalence (between 4.9 and 77.7%) than reported in our study. In South America, there are reports of *Bartonella* spp. in vector species of wild ungulates (68, 90) but not in their blood samples (68). In Chile, during the last decade, there have been reports of the presence of *Bartonella* spp. in cats, dogs, minks, and bats (23–26, 28, 91, 92). *Bartonella henselae* in pudu was similar to *B. henselae* reported in small Indian mongooses in the Caribbean (93) and cats from Brazil (94) and Paraguay (27). *Bartonella* sp., related to those reported infecting ruminants, such as *B. schoenbuchensis* [CP019789, HG977197 (95)], *B. chomeli* (KM215718) (96), *B. melophagi* (JF834886) (97), *B. bovis*, and *B. capreoli*, was also detected in a pudu in this study. More screening will be necessary to confirm the role of pudu in the epidemiology of this infectious agent and its impact on animal health.

Hemoplasma bacteria have been extensively studied in wild and domestic carnivores in Chile during the last decade. Darwin foxes (*Lycalopex fulvipes*) present a high prevalence of *M. haemocanis* causing enzootic and asymptomatic infections (18, 19) that could be a source of infection for pudu since both share the same habitat within the Los Lagos region. Hemotropic *Mycoplasma* spp. have been recently reported in llamas (12.8%) and alpacas (6.3%) (*Candidatus Mycoplasma haemolamae*) (31) and *Mycoplasma ovis*-like in free-living pudu in southern Chile (14%) (10). Molecular screening of hemotropic *Mycoplasma* spp. in sheep, livestock, and native (huemul) and exotic ungulates (wild boars, red deer) in the Los Lagos District is recommended to understand the epidemiology of these infectious agents and the possible role of pudu as a host. It is also recommended to evaluate the pathogenicity of *Mycoplasma ovis*-like in pudu.

For the first time in pudu, the finding of *B. henselae* and *C. burnetii*, both zoonotic pathogens, could be relevant to public health. Both *B. henselae* and *C. burnetii* are pathogens related to occupational diseases, with evidence of health risks for those working with infected species through occupational exposure in rehabilitation centers, breeding centers, and zoological parks (98, 99), representing an important factor to consider in medical and management practices with this animal species.

## 5 Conclusion

This study expands the knowledge of bacteria with zoonotic potential carried by pudu. *Mycoplasma ovis*-like was confirmed in pudus, while *Bartonella* spp., *Bartonella henselae*, and *C. burnetii* were described for the first time in South American ungulates. The results of this study suggest an anthropic impact on wildlife species with domestic species interacting epidemiologically with pudus in their natural habitats. Further research will be necessary to evaluate the potential role of pudu as a host and reservoir of infection, and identify the sources for disease transmission among humans and wild and domestic animals.

## Data availability statement

The datasets presented in this study can be found in online repositories. The names of the repository/repositories and accession number(s) can be found below: https://www.ncbi.nlm.nih.gov/nuccore; OQ162290, OQ137267, OQ162291.

## Ethics statement

Ethical approval was not required for the study involving animals in accordance with the local legislation and institutional requirements because we only use samples from Frozen Banks of the rehabilitation centers and zoos. We don't have involved in any managements animals procedure.

## Author contributions

EH-H: conceptualization, supervision, funding acquisition, investigation, resources, data curation, writing—original draft preparation, and writing—reviewing and editing. PS-G: methodology, investigation, resources, data curation, writing—original draft preparation, and writing—reviewing and editing. JC, CV, FV, and IK: resources and data curation. SC and CO: resources. DM-A: writing—original draft preparation, and writing—reviewing and editing. MO and NC: methodology and investigation. AG: investigation and writing—reviewing and editing. AM: conceptualization, supervision, funding acquisition, methodology, investigation, writing—original draft preparation, and writing—reviewing and editing. All authors contributed to the article and approved the submitted version.
